# Satisfaction of Female Patients with Health Care Services at the Peri-urban Community Health Centre in Islamabad

**DOI:** 10.7759/cureus.3101

**Published:** 2018-08-04

**Authors:** Saba Savul, Zahid Naeem, Sajida Naseem

**Affiliations:** 1 Community and Family Medicine, Shifa College of Medicine, Islamabad, PAK; 2 Community and Family Medicine, Shifa International Hospital, Islamabad, PAK

**Keywords:** patient satisfaction, community health centre, primary health care, islamabad, pakistan

## Abstract

Objective

To evaluate the satisfaction levels of female patients with the availability and quality of health care services provided at the Community Health Centre (CHC) in Nurpur Shahan, a deprived peri-urban locality in Islamabad.

Methods

This cross-sectional study was carried out among 346 randomly selected female patients who attended the CHC in Nurpur Shahan from January to March, 2018. Data was collected by face to face interviews using a pre-tested self-designed questionnaire. Data was analysed using SPSS version 23 (IBM Corp., Armonk, NY, USA).

Results

Female patients were largely satisfied with the behaviour (96%) and competence (97.1%) of doctors, the attitude of the paramedical staff (93.6%), quality of medicines (93.6%) and basic facilities at the CHC including drinking water, bathrooms, and cleanliness. Patients had mixed satisfaction levels with various aspects of antenatal services, post-natal care, immunization services as well as the location of CHC (58.7%), availability of prescribed medicines (67.1%) and affordability of laboratory tests at the centre (63.3%). Major areas of discontent were health education regarding breastfeeding and immunization, the average waiting time to see a doctor, the waiting area, and family planning services.

Conclusion

Although patient satisfaction levels with certain health care services were good, there is considerable room for improvement in other aspects of provided services at the CHC.

## Introduction

Pakistan is a low income, developing country which lacks resources and good infrastructure. Government expenditure on health is minimal in terms of GDP. For effective implementation of primary health care services, certain pre-requisites must be met including suitable premises, appropriate equipment, competent workforce, adequate technology and medicines, sustainable funding systems, community participation and inter-sectorial collaboration [1]. Community health centres (CHCs) aim to provide comprehensive, assessable, affordable and equitable health services for everyone without any discrimination [2-3]. They play a vital role in Pakistan to reach out to vulnerable, medically underserved and under privileged communities [4].

Nurpur Shahan is a deprived peri-urban area located in the suburbs of Islamabad. The residents of Nurpur Shahan live in extreme poverty and lack basic amenities such as health care facilities, water, and sanitation. The nearest medical facility available to the residents is a community health centre (CHC) which was established by Shifa Tameer-e-Millat University in collaboration with the Rotary Club Cosmopolitan. A significant proportion of patients who visit this CHC are women seeking general medical attention, reproductive health services, antenatal and post-natal care. Women in Pakistan do not enjoy the same rights as men. The quality, accessibility, and utilization of health services by women belonging to the low socio-economic class is inadequate and inequitable [5].

Research conducted in developing countries to determine female patient satisfaction in primary health care facilities demonstrates a complex myriad of issues. In Pakistan, a study conducted in 2016 to determine female patient satisfaction at a CHC in Mardan District of Khyber Pakhtunkhwa province found that study participants were largely satisfied with medical and obstetric care, medicine availability, laboratory tests, ultrasound facility, staff interaction, staff cooperation and premises [4]. However, 50% of women were not satisfied with the provided health care services in primary health care centres in Punjab. The major reasons were the distant location of health centres, lack of functional equipment, inadequate supplies, inconvenient working hours, indifferent attitudes and non-availability of staff [6].

In India, female patients were highly satisfied with general health services, antenatal and immunization services but less so with post-natal services at CHCs [7-8]. Conversely, in 14 Indian primary health care centres, women rated the quality of ante-natal care and child-related services as mediocre [9]. In another Indian study, good satisfaction score was given to services of healthcare professionals but feedback was put forward for the reduction in waiting times, separate laboratory in all centres, privacy, spaciousness, separate waiting area for male and female patients with fan and lighting facilities [10].

In Nigeria, the level of satisfaction with health services and competence of health care personnel was good [11-12] and low cost of services, use of local language [13], staff attitude and interaction [13-14] were highlighted as positive factors. Long waiting times, poor sanitary facilities [11-12] and lack of privacy [15] were major areas of discontentment.

Two Egyptian studies established that the issues of dissatisfaction for clients were the registration process, waiting area, waiting time, relation with nurses and medical assistant staff [16], the location of a health centre, health education methods and explanation of problems by physicians [17]. In Iraq, the areas of concern were crowdedness, unsuitable location, unavailability of ultrasound, low number of doctors and female staff [18], poor family planning and postnatal services [19] and lack of health education opportunities [19-20].

The aim of our study was to determine the satisfaction levels of female patients with the availability and quality of health care services provided at the community health centre in Nurpur Shahan. It is anticipated that the results of this study will lead to a better understanding of the health care needs for female patients and emphasize potential areas for improvement of health services at the CHC in Nurpur Shahan.

## Materials and methods

A cross-sectional survey was conducted from January to March, 2018 for a total period of three months to evaluate the satisfaction of female patients with the availability and quality of health care services provided at the CHC in Nurpur Shahan, Islamabad. Female patients were selected by systematic random sampling by including every 11th patient in the study. A sample size of 346 patients was calculated using Epi Info using the following information: total number of patients in a month as 3500, confidence intervals as 95%, expected frequency as 50% [6] with 5% acceptable margin of error. All female patients above the age of 18 years and who were able to understand Urdu were included in the study. Since the working hours of the CHC are from 9:00 am to 2:00 pm during which most men are at work, women constitute the bulk of patients attending the centre. All male patients and children visiting the CHC, and female patients who refused to participate in the study were excluded.

The study commenced after approval from the Institutional Review Board and Ethics Committee of Shifa International Hospital (Reference number: 945-220-2018). Written informed consent was obtained from every study participant. Each participant was required to sign or give thumb impression on the consent form. It was made clear to the study participants that taking part in the study was voluntary and they had the right to withdraw from the study at any time. Data was collected by a self-designed questionnaire consisting of a socio-demographic profile of the study participants and domains of patient satisfaction including availability and quality of health care and other services provided at the CHC. The questionnaire was translated into Urdu and then translated back into English to ensure its reliability by two bilinguals. The questionnaire was interviewer administered. It was pretested on study participants to ensure its validity. Data was analysed using SPSS version 23 (IBM Corp., Armonk, NY, USA). Descriptive statistics including frequency and percentage were calculated for qualitative variables.

## Results

The research involves the responses of 346 female patients from the CHC in Nurpur Shahan, Islamabad. Table 1 depicts the sociodemographic characteristics of study participants.

**Table 1:** Socio-demographic characteristics of study participants CHC: Community Health Centre

Characteristic	Frequency	Percentage
Age (years)		
18-28	208	60.1
29-39	100	28.9
40-50	23	6.6
> or = 51	15	4.3
Education Level		
Illiterate	182	52.6
Primary	74	21.4
Secondary & above	90	26.0
Marital Status		
Single	16	4.6
Married	327	94.5
Widowed	3	0.9
Employment Status		
Employed	17	4.9
Unemployed	329	95.1
No. of rooms in house		
One	52	15.0
Two	152	43.9
Three or more	142	41.0
Total monthly income (PKR)		
<5,000	44	12.7
5,000-10,000	104	30.1
>10,000	198	57.2
Visit to CHC in last 3 months		
Once	145	41.9
Twice	103	29.8
More than twice	98	28.3
Main reason for visit to CHC		
Medical	188	54.3
Gynaecological	34	9.8
Obstetrical	24	6.9
Paediatric / Immunization	89	25.7
Family Planning	10	2.8

Table 2 represents patient satisfaction with doctors and paramedical staff at the CHC

**Table 2:** Satisfaction with doctors and paramedical staff

	Frequency	Percentage
Average waiting time for doctor		
10-20mins	3	0.9
20-30mins	55	15.9
>30mins	288	83.2
Proper examination done by doctor		
Yes	336	97.1
No	1	0.3
Not sure	9	2.6
Privacy maintained during the examination		
Yes	336	97.1
No	4	1.2
Not sure	6	1.7
Satisfaction with attitude of doctors		
Yes	332	96.0
No	7	2.0
Not sure	6	1.7
No response	1	0.3
Satisfaction with attitude of paramedical staff		
Yes	324	93.6
No	17	4.9
Not sure	3	0.9
No response	2	0.6

Table 3 displays patient satisfaction levels with general facilities at the CHC.

**Table 3:** Satisfaction with general facilities at the Community Health Centre (CHC)

Satisfaction with facilities	Frequency	Percentage
Drinking water		
Yes	248	71.7
No	43	12.4
Not sure	55	15.9
Proper waiting area with chairs		
Yes	274	79.2
No	72	20.8
Separate waiting area for male and female patients		
Yes	4	1.2
No	335	96.8
Not sure	7	2.0
Fan/heater		
Yes	250	72.3
No	51	14.7
Not sure	45	13.0
Bathrooms		
Yes	259	74.9
No	16	4.6
Not sure	67	19.4
No response	4	1.2
Electricity		
Yes	260	75.1
No	27	7.8
Not sure	59	17.1
Cleanliness of CHC		
Yes	286	82.7
No	60	17.3
Location of CHC		
Yes	203	58.7
No	142	41.0
No response	1	0.3

Satisfaction with quality and availability of medicines

A substantial majority of 324 patients (93.6%) were satisfied with the quality of available medicines. Two hundred and thirty-two (67.1%) patients complained that they were unable to obtain all prescribed medicines from the CHC and were asked to purchase it from the market.

Satisfaction with antenatal and postnatal services

In our study, 204 (59%) and 215 (62.1%) patients were satisfied with counselling and examination respectively, whereas 115 (33.2%) patients stated that they were given information about breastfeeding.

Satisfaction with immunization services

A sizable number of 256 (74%) patients stated that a proper entry was made in the immunization card. A hundred and one (29.2%) patients claimed that they were given information regarding benefits and side effects of vaccines whereas 236 (68.2%) patients affirmed that vaccines were available on their visit. For proper management done in case of a reaction or side effect from a vaccine 170 (49.1%) patients answered “yes”.

Satisfaction with laboratory tests

The research results revealed that 249 (72%) patients were satisfied with the tests performed at the centre and 219 (63.3%) patients found the test charges affordable.

Satisfaction with family planning services

In this study, 78 (22.5%) patients stated that they were given advice on spacing methods and 58 (16.8%) declared that they were informed about contraceptive methods. Forty-six (13.3%) patients were aware regarding availability of free condoms at the CHC, 66 (19.1%) patients knew about free oral contraceptive pills, and 30 (8.7%) patients voiced that free IUCDs were available at the centre.

Patient suggestions for improvement in CHC services

In the CHC, the working hours are from 9:00 am to 2:00 pm. Patients provided feedback that the opening hours of the CHC should be increased. Suggestions were put forward for reduction in waiting times and provision of separate waiting area for male and female patients. Patients stressed that all prescribed medicines should be available at CHC and tests should be affordable or free. It was proposed that more health facilities and equipment should be available at the CHC including emergency services, facilities for delivery, X-ray, electrocardiogram (ECG), and ultrasound.

## Discussion

The study results demonstrate varying levels of satisfaction with health care services provided at the CHC at Nurpur Shahan, Islamabad. This is in sharp contrast to a recent study in which female patients gave high satisfaction scores to health care services available at CHC in Khyber Pakhtunkhwa, Pakistan [4]. In our study, female patients were highly satisfied with the attitude and competence of doctors at the CHC. However, the average waiting time to see the doctor was exceedingly long as established by other studies in developing countries [10-12,16].

Good satisfaction scores were given to general basic facilities at the CHC such as drinking water, electricity, bathrooms and cleanliness. However, 142 (41%) patients desired that the centre was nearer to their homes as they had to travel a long distance to reach there. Non-feasibility of location was also highlighted in a study carried out in multiple primary health care centres in Punjab, Pakistan [6]. A pressing need for a separate waiting area for men and women was put forward by the study participants which is analogous to the findings of a study conducted in India [10].

Our study results demonstrate that there is considerable room for improvement in antenatal and postnatal health services. These findings are corroborated by studies carried out in Indian primary health care centres where ante natal [9] and post-natal services [7-8,19] had low satisfaction scores. Counselling regarding breastfeeding was a consistently neglected area according to the results of this study. Female patients should be given complete information regarding breastfeeding including its benefits for mothers and infants.

Satisfaction with family planning services ranked very low with female patients, a finding similar to another study conducted in Iraq [19]. A large number of patients were not given information about contraceptive methods and were ignorant of the availability of contraceptive methods at the CHC. This is supported by the fact that only 10 patients visited the CHC exclusively seeking family planning services. Information regarding different family planning and contraceptive methods must be disseminated to the patients to increase utilization of family planning services. This is very important in the context of Pakistan where the total fertility rate is high and the contraceptive prevalence rate is very low.

Some areas of immunization services had low satisfaction rates, especially counselling regarding the benefits and side effects of vaccines (29%). Although the quality of available medicines at the CHC was rated highly, all prescribed medicines were not available at the CHC and patients were asked to buy them from the market. Since the majority of the patients visiting the CHC are poverty stricken, they are unable to purchase medicines as a result of which their medical treatment and health suffers. Another critical concern was that only 63% patients found the test charges affordable and articulated that the costs of tests should be lowered or free as patients generally forgo tests which they cannot afford which is detrimental to their health.

Since the study sample is limited to female patients at CHC in Nurpur Shahan, the results of the study are not representative of the whole population and cannot be generalized to other CHCs in Pakistan.

## Conclusions

Although satisfaction levels with some health care services were good, there is considerable room for improvement in other aspects of provided services at the CHC in Nurpur Shahan, Islamabad. Factors which should be given due consideration are health education regarding immunization, family planning and breastfeeding, augmenting antenatal and postnatal services, lowering costs of tests, increasing the available medicines at the CHC, reducing waiting times to see doctors and providing a separate waiting area for women to enhance the satisfaction of health care services utilized by the female patients.
